# Immunomodulatory roles of selenium nanoparticles: Novel arts for potential immunotherapy strategy development

**DOI:** 10.3389/fimmu.2022.956181

**Published:** 2022-07-26

**Authors:** Gengshi Chen, Fen Yang, Shuhao Fan, Hua Jin, Kangsheng Liao, Xuemeng Li, Gan-Bin Liu, Jing Liang, Junai Zhang, Jun-Fa Xu, Jiang Pi

**Affiliations:** ^1^ Guangdong Provincial Key Laboratory of Medical Molecular Diagnostics, The First Dongguan Affiliated Hospital, Guangdong Medical University, Dongguan, China; ^2^ Institute of Laboratory Medicine, School of Medical Technology, Guangdong Medical University, Dongguan, China; ^3^ Guangdong Provincial Key Laboratory of Medical Molecular Diagnostics, Institute of Pathogenic Biology and Immunology, School of Basic Medicine, Guangdong Medical University, Dongguan, China; ^4^ Department of Respiration, Dongguan 6th Hospital, Dongguan, China

**Keywords:** Selenium nanoparticles, immunotherapy, macrophages, NK cells, T cells

## Abstract

Current chemotherapy strategies used in clinic appear with lots of disadvantages due to the low targeting effects of drugs and strong side effects, which significantly restricts the drug potency, causes multiple dysfunctions in the body, and even drives the emergence of diseases. Immunotherapy has been proved to boost the body’s innate and adaptive defenses for more effective disease control and treatment. As a trace element, selenium plays vital roles in human health by regulating the antioxidant defense, enzyme activity, and immune response through various specific pathways. Profiting from novel nanotechnology, selenium nanoparticles have been widely developed to reveal great potential in anticancer, antibacterial, and anti-inflammation treatments. More interestingly, increasing evidence has also shown that functional selenium nanoparticles can be applied for potential immunotherapy, which would achieve more effective treatment efficiency as adjunctive therapy strategies for the current chemotherapy. By directly interacting with innate immune cells, such as macrophages, dendritic cells, and natural killer cells, selenium nanoparticles can regulate innate immunity to intervene disease developments, which were reported to boost the anticancer, anti-infection, and anti-inflammation treatments. Moreover, selenium nanoparticles can also activate and recover different T cells for adaptive immunity regulations to enhance their cytotoxic to combat cancer cells, indicating the potential of selenium nanoparticles for potential immunotherapy strategy development. Here, aiming to enhance our understanding of the potential immunotherapy strategy development based on Se NPs, this review will summarize the immunological regulation effects of selenium nanoparticles and the application of selenium nanoparticle-based immunotherapy strategies. Furthermore, we will discuss the advancing perspective of selenium nanoparticle-based potential immunotherapy as a kind of novel adjunctive therapy to enhance the efficiency of current chemotherapies and also introduce the current obstacles for the development of selenium nanoparticles for potential immunotherapy strategy development. This work is expected to promote the future research on selenium nanoparticle-assisted immunotherapy and finally benefit the more effective disease treatments against the threatening cancer and infectious and chronic diseases.

## Introduction

Cancer, an uncontrollable, malignant disease, has been a significant threat to human beings causing millions of deaths worldwide every year. The low efficiency and strong side effects of the current chemotherapy remain substantial challenges for effective cancer therapies (1). By boosting the body’s natural defenses, immunotherapy has emerged as a type of novel cancer treatment strategy that helps your immune system fight tumor. However, the low efficiency of current PD1/PD-L1 therapies against cancer is widely restricted to show only 10%–30% effectiveness, which therefore requires the development of new immunotherapy methods. With deadly disorders caused by microorganisms, infectious diseases have been highlighted as the most urgent global health issue. The increasing drug-resistant mutants continually weaken the current therapies, which therefore also required the developments of new treatment methods. Immunotherapy manipulates different immunological components to target and eliminate pathogens or diseased host cells to offer protection against disease or alleviate symptoms without drug-resistant issues and is now expected to provide more effective control of infectious diseases. Chronic diseases, such as diabetes and atherosclerosis, pose a massive threat to human health. Especially during the pandemic of COVID-19, more and more studies emphasized that patients of chronic disease with underlying medical conditions were at a higher risk of developing severe symptoms (2). Currently, although these methods, including surgery, hormone therapy, and conventional medicine, were being used, side effects such as statin-related muscle symptoms (SAMs) usually occurred (3). At such a specific position, the introduction of immunotherapy into chronic disease for more effective treatments becomes an urgent issue. However, the current immunotherapy strategies have been still facing some important issues, such as the inability to predict treatment efficacy and patient response, the need for additional biomarkers, the development of resistance to immunotherapy, the lack of clinical study designs, and high treatment costs. Thus, it needs more concerns to develop more effective immunotherapy strategies.

Selenium, an essential trace element to support bodily physiological function and processes, is considered an essential trace element of fundamental importance for human health (4). Insufficient supplementation of this element results in the increased risk of developing many chronic degenerative diseases, which therefore requires selenium supplementation in populations at high risk of low selenium intake (5). Excess selenium (sodium selenite) treatment also has a negative effect on health by exhibiting toxicity and causing increased lipid peroxidation due to the strong oxidative stress induced by high doses of selenium (6). Several studies have indicated that selenium deficiency may be detrimental in the development and therapy of different kinds of viral infection diseases (7). It was also found that selenium deficiency was associated with worse outcomes in COVID-19 patients, and selenium levels in COVID-19 patients were lower than in healthy individuals (8, 9). Thus, it could be speculated that cautious selenium supplementation in COVID-19 patients may be helpful to prevent disease progression (10); however, more works are still needed to further confirm it.

Physiologically active selenium is incorporated into proteins in the form of selenocysteine, a derivative of the amino acid cysteine, in which it replaces sulfur in the thiol group (-SH) (11). Selenium is also a component of selenomethionine, which is also widely involved in the construction of certain enzyme proteins. Thus, selenium is a component of many key enzymes for life events, including glutathione peroxidase (GPx) and thioredoxin reductase (TrxR). Due to the critical roles of selenoproteins in human health, selenium directly participates in human metabolism in the form of GPx, contributing to the control of H_2_O_2_, fatty acid hydroperoxides, heavy metal detoxification, and regulation of the immune and reproductive systems as well (12, 13). Besides GPx, TrxR, an enzyme in defense against oxidative damage, is also controlled by selenium and selenium deficiency could lead to a decrease in the body’s antioxidant defense, the development of oxidative stress, inflammation, and apoptosis in various cell types (14). As part of these key enzymes can catalyze oxidation–reduction reactions for many metabolic changes, selenium can therefore protect cells against the harmful effects of free radicals by showing antioxidant effects.

Selenoproteins are also involved in the development and regeneration of muscle tissue, and the selenium deficiency or selenoprotein N dysfunction is associated with the muscle disorders (15). In addition, selenium is also involved in the recovery of ascorbic acid from its oxidized metabolites, participating in the processes of DNA synthesis and apoptosis (11). Moreover, increasing evidence has indicated that selenium plays very important roles in cancer inhibition (16). Selenium can inhibit the division and growth of several neoplastic cells and can also selectively protect normal cells from cellular stress (17, 18), while the mechanisms are still needed to be further explored. Kieliszek et al. have proposed several hypotheses concerning the anticancer activity of selenium, including the conformational alterations of proteins through sulfhydryl group oxidation by selenium components, which can affect the ability of proteins to weaken the activity of enzymes involved in the metabolism of cancer cells (19). For example, human fibrinogen sodium selenite can inhibit protein disulfide exchange reactions, thus preventing the formation of a hydrophobic polymer termed parafibrin (19). Parafibrin can specifically form a protein coat around tumor cells that is completely resistant to degradation induced with lymphocyte protease (20), which therefore supports the hypothesis that selenium is helpful to inhibit the resistance of cancer cells to the degradation and is induced with lymphocytes (19). Additionally, selenite may directly activate NK cells, as well as inhibit angiogenesis without an undesirable decrease in the oxidative potential of the cellular environment, which therefore indicates that sodium selenite might become a drug of choice for different types of cancer (21).

However, limited to its narrow safety dosage, excess selenium could cause toxic symptoms, including nausea, nail discoloration, and vomiting (22). How to actively apply the biological functions of selenium for human health and disease control remains a challenge. Profiting from nanotechnology, selenium nanoparticles (Se NPs) have recently been proved to show stronger biological activity and lower toxicity than the traditional selenium compounds. Bare Se NPs quickly tend to aggregate into dark clusters due to their high surface energy, which results in the low bioactivity (23, 24). By introducing different kinds of stabilizers into the system, stable Se NPs can be obtained by manipulating the selenium atom into nanoscale particle aggregates. The typical stabilizers of Se NPs can be biological molecules with some active groups, such as amino groups, hydroxyl groups, and carboxylate groups. For example, polysaccharides were widely utilized as stabilizers for Se NPs, which could significantly enhance the stability of Se NPs in an aqueous solution (25). Stable Se NPs were utilized more efficiently in anticancer or anti-infection treatments compared with inorganic and organic selenium compounds (26, 27). Benefiting from its biocompatibility, Se NPs could exert various biological functions including cell apoptosis, autophagy, and drug delivery (28). Wu et al. concluded that Se NPs had great ability to regulate oxidative stress and immunity (29). Recent studies indicated that Se NPs could also serve as therapeutic agents due to their potential for anti-atherosclerotic effect and alleviating histological damage caused by diabetes (30, 31). These previous results collectively suggest that Se NPs have widely biological application by combining the advantages of the biological activity of selenium and the nanoscale properties.

More importantly, Se NPs have also been proved to show excellent potentials for immunotherapy, which significantly improve the treatment efficiency of cancer and infectious and chronic diseases by regulating different immune cell functions. Herein, aiming to enhance our understanding of the potential immunotherapy strategy development based on Se NPs, we summarized the recent exciting progress for the immunomodulatory roles of Se NPs by targeting different kinds of immune cells. Additionally, we discussed the potential of Se NPs for more effective disease treatment as a novel adjunctive therapy strategy, which will be beneficial for immunotherapy strategy developments against cancer and infectious and chronic diseases in the future.

## Basic biological function of Se NPs

Se NPs have attracted increasing attention from nanotechnology, engineering, biological, and medical fields due to their excellent properties. Moreover, the basic biological properties of Se NPs would finally contribute the potential of Se NPs for immunotherapy. Hence, the noticeable biological functions of Se NPs, especially its application in immunotherapy, are both discussed in this section.

## Se NPs regulate oxidative stress in normal tissues and cells

Oxidative stress is an imbalance between free radicals and antioxidants in the body, which partially determines the health status. Reactive oxygen species (ROS) are by-products of metabolism, widely known as a double-edged sword in the body to regulate the oxidative stress. ROS plays a critical role in cell signaling and homeostasis, whereas excess doses of ROS would cause damage to membranes, lipids, nucleic acids, and organelles. Selenium is involved in the metabolism of hydrogen peroxide and lipid hydroperoxides because it constitutes an integral part of some enzymes, which protect cells from the noxious effects of free radicals formed during oxidation processes (12). However, it is also widely accepted that high doses of selenium (sodium selenite) could cause oxidative stress in organisms, thereby increasing the process of lipid peroxidation and indicating that excessive intracellular accumulation of selenium is toxic to organisms (6). Thus, the regulatory effects of selenium on oxidative stress make it a potential candidate for treatment of oxidative stress associated disorders.

Taking the advantages of selenium properties, Se NPs could commonly act as an antioxidant factor in normal tissues and cells, which controlled ROS levels to regulate some important signaling events (32, 33). It is reported that stabilized Se NPs could effectively alleviate PAT-induced excessive production of intracellular ROS, the decline of glutathione peroxidase activity, and the suppression of cell viability (34). Moreover, Se NPs could also ameliorate patulin-induced oxidative damage to the liver and kidney mainly owing to its alleviation effect against ROS. Although cisplatin is still one of the most effective chemotherapy agents for human cancers, its clinical use is limited by serious side effects, especially nephrotoxicity that is controlled by cisplatin-induced oxidative stress. Li et al. reported a kind of simple method for the functionalization of Se NPs by self-assembly of 11-mercapto-1-undecanol (Se@MUN), which showed a much higher cellular uptake in human normal cells by comparing it with SeNPs (35). Se@MUN was found to significantly prevent the cisplatin-induced overproduction of intracellular ROS in proximal tubular cells, which therefore regulate the following apoptosis signaling to prevent cisplatin-induced apoptosis. Their findings suggest that Se@MUN is a promising selenium species with potential application in the prevention of cisplatin-induced renal injury. These studies suggested that Se NPs could serve as a promising species with potential application in the prevention of chemotherapy drug-induced ROS overproduction to regulate the oxidative stress in normal cells, which would alleviate the side effects of chemotherapy.

## Se NPs show anti-inflammatory activities

The role of selenium in inflammation has been elucidated, as it acts as a regulatory agent mainly in form of selenoproteins and incorporation (36). For example, selenoprotein S (Sel S) is conduced to wound healing by impacting the inflammation phase by regulating the inflammatory response (37). An investigation on children with systematic inflammation also indicated that increased plasma selenium was closely related to better outcomes (38). Inheriting the merit of selenium, Se NPs also exert excellent properties to control inflammation (39).

Vancomycin (VCM) is commonly applied to treat multiresistant Gram-positive bacteria, but its clinical application is limited due to its nephrotoxicity. Mehanna et al. investigated the ability of Se NPs to protect against VCM-induced nephrotoxicity in rats. The obtained results indicated that Se NPs could efficaciously reduce VCM-induced pro-inflammatory factors and oxidative molecules such as lipid peroxidation product malondialdehyde (MDA), inducible nitric oxide synthase (iNOS), nitric oxide (NO), tumor necrosis factor-α (TNF-α), and kidney injury molecule 1 (KIM-1), as well as downregulating pro-apoptosis molecules to protect kidneys (40). Xiao et al. found that Se NPs alleviated not only vascular endothelial dysfunction, as evidenced by the increase in serum nitric oxide level and the decrease in aortic adhesion molecule expression, but also vascular inflammation, as evidenced by the decrease of macrophage recruitment as well as the inhibition of the NF-κB signaling pathway and iNOS (41). Amani et al. also introduced Se NPs for targeted stroke therapy through modulation of inflammatory and metabolic signaling (42), which provided a promising treatment strategy for cerebral stroke based on the anti-inflammatory effects of Se NPs. These attractive properties to reduce pro-inflammatory factors and oxidative stress could therefore serve as novel treatments against inflammatory disorders.

## Se NPs serve as drug and gene carriers

Nanomedicine has been high-profile for decades as it remolds traditional therapeutic strategies, especially for their roles in drug or gene delivery. Owing much to their high selectivity and biocompatibility, Se NPs reveal excellent properties as drug and gene carriers to highly improve their therapeutic efficiency. The non-targeting effects of chemotherapy agents against cancer cells remain the most challenge issue for the low efficiency and high toxicity of cancer chemotherapy. Xia et al. loaded doxorubicin (DOX) into RGDfC (cyclic peptide)-modified Se NPs and precisely targeted the non-small cell lung cancer A549 cells (43). Due to the specific targeting effects of RGDfC against cancer cells, RGDfC-Se@DOX successfully delivered DOX to cancer cells and reduced the toxicity of DOX that is brought to normal cells. Additionally, Xia et al. also utilized galactose-modified Se NPs to deliver DOX to the HepG2 cancer cell, indicating excellent properties of Se NPs as drug carriers (44).

Recent studies have also proved that Se NPs are capable of delivering different kinds of genes into cells. Wang et al. capitalized on RGDfC-modified Se NPs by creatively applying them to carry siRNA (45). According to their research, Se NPs showed great capacity and remarkable ability to protect siRNA from degradation and could selectively deliver siRNA into the target cells with high delivery efficiency, which indicated that Se NPs are excellent siRNA carriers in gene delivery. Likewise, Se NPs could also be applied as a carrier for mRNA delivery. Singh et al. modified Se NPs with chitosan (CS), polyethylene glycol (PEG), and lactobionic acid (LA), forming a stable and functional delivery system (46). These loaded Se NPs accelerated the internalization and controlled the release of mRNA while they prevent RNA degradation led by nucleases, successfully realizing mRNA delivery to the HepG2 cancer cell. These results indicated that Se NPs are ideal carriers for drug and gene delivery, which can contribute to the further immunotherapy application of Se NPs.

## Se NPs induce apoptosis

High ROS levels could induce apoptosis by activating the tumor-suppressor protein p53. While p53 induced DNA repair in low-stress conditions, it upregulates pro-apoptotic factors and downregulates pro-survival proteins under severe stress (33). Although selenium plays important roles in oxidative stress regulation by their roles in selenoproteins, high doses of sodium selenite could cause oxidative stress in organisms, thereby increasing the process of lipid peroxidation (6). Sodium selenite treatment has been widely proved to cause a significant increase in intracellular reactive oxygen species (ROS) levels, which therefore further promote the apoptosis of cells (47, 48).

Similarly, Se NPs could serve as antioxidants in normal cells but could dramatically increase ROS production in cancer cells, which might be attributed to the abnormal metabolism status of cancer cells. Huang et al. found that Se NPs could successfully induce ROS overproduction in cancer cells and trigger apoptosis through the p53 pathway (49). What is more, Se NPs could also induce apoptosis by impacting mitochondria. Firstly, Se NPs that have been internalized into the cells could cause ROS accumulation in the cells. Then, the overproduction of ROS stimulated mitochondrial permeability transition pore opening. It led to the release of cytochrome c, which disrupts the electron transport chain, resulting in mitochondrial damage-leaking cytochrome c and apoptotic protease activating factor 1 (Apaf-1) and pro-caspase 9 forming a complex and further developing apoptosis (50, 51). Additionally, ROS produced by Se NPs act on p53 and AKT signaling pathways (52). Our previous work also indicated that Se NPs could induce cancer cell apoptosis by inhibiting EGFR-mediated PI3K/AKT and Ras/Raf/MEK/ERK pathways after loading with oridonin (53). What is more, it is reported that Se NPs could trigger MAPK activation and the caspase-3 signal pathway, eventually inducing apoptosis of tumor cells (54, 55). These results suggest that Se NPs could serve as strong apoptosis-induction agents, which therefore shows the potential of Se NPs as novel antitumor agents by inducing cancer cell apoptosis.

## Se NPs promote autophagy

Autophagy is characterized by nuclear fragmentation and cell shrinkage, contributing to updating organelles and materials for cell survival (56). It is reported that dysfunctional autophagy might lead to neurodegenerative diseases (57). Tumors could be suppressed by autophagy through impacting the developmental stage and tumor type, while dysfunction of autophagy impaired tumor inhibition and caused tumorigenesis (58). In the dynamic, multistep progress of autophagy, the ULK complex and PI3K III complex (PI3KIII, Beclin-1, Atg14/Barkor, Vps15, and Ambra-1) initiated and other ATG proteins were recruited to form the phagophore. Subsequently, the microtubule-associated protein light chain 3 (LC3) protein gathered to the phagophore, allowing the autophagosome to mature (59).

Autophagic cell death also occurred after selenite exposure in cancer by regulation of different pathways (60, 61), suggesting an alternative mechanism for the potential therapeutic properties of selenium. Considering this, researchers applied Se NPs in cancer cells and found that the autophagy induced by Se NPs participates in antitumor activities through elevating Beclin-1-related signaling pathways and downregulating p62, the protein which is inversely related to autophagy (62–64). Mi et al. loaded silymarin on Se NPs, confirming that autophagy was triggered in gastric cancer cells, along with a significant increase in Beclin1 and LC3 II (65). Their results further demonstrated that autophagy was activated by Se NP treatment *via* inhibiting the PI3K/AKT/mTOR pathway.

In some studies, nevertheless, Se NPs have been found to inhibit autophagy in order to exert cytotoxicity in cancer cells. Here, it is necessary to notice the dual role of autophagy in cancer development. Some tumors draw the help of autophagy to overcome hypoxia and starvation and eventually survive and proliferate (66). Cui et al. have observed that p62 and Beclin-1 were increased after treating Se NPs in cancer cells for 12 h, suggesting that the late phase of autophagy was inhibited by Se NPs (67). Further experiment showed that Se NPs interfered the function of lysosomes and alkalized lysosomal environment, blocking the late phase of autophagy. Interestingly, induction of autophagy by Se NPs has also been proved in infectious diseases. Our previous work demonstrated that Se NPs promoted intracellular Mtb clearance by enhancing autophagy, providing a novel method to combat Mtb (68). All the evidence above strongly indicated that Se NPs promoted autophagy.

## Immunotherapy application of Se NPs

The emergence of the immunotherapy strategy dramatically improved the treatment efficiency of the current chemotherapy. However, due to the limited efficiency of the existing immunotherapy methods, increasing attention is paid to the exploration of novel therapeutic strategies with nanoparticles, aiming to promote immunotherapy. Dietary supplementation with optimum levels of selenium was proved to exert beneficial effects on host immune response to necrotic enteritis and reduced the negative consequence of necrotic enteritis-induced immunopathology (69). Moreover, the increase in selenium (sodium selenite) intake could improve immune function and poliovirus handling in adults with a marginal selenium status (70). These results collectively suggested that selenium plays important roles in immune regulation, which therefore demonstrate the potential of Se NPs for immune regulations.

## Se NPs program macrophage immune responses

As the first defensive line of the immune system, macrophages, originating from the mononuclear phagocyte system (MPS), are one of the most essential parts of innate immunity. Compared with other kinds of cells, macrophages are highly heterogenous cells that can rapidly change their function in response to local microenvironmental signals. Thus, macrophages adopt context-dependent phenotypes that either promote or inhibit host antimicrobial defense, antitumor immune responses, and inflammatory responses in response to different disease situations (71). In recent decades, more and more researchers have been attracted by the distinct immune functions of macrophages and focused on the immunotherapy strategy developments targeting macrophages. Up to now, there are still challenges in activating macrophage effectively, as a result of binding signal regulatory protein alpha (SIRPα) to CD47 on cancer cells. Meanwhile, tumor-associated macrophage (TAM) is easily polarized to M2 type by tumor, which aggravates disease progression (72). Therefore, most macrophage-immunotherapeutic strategies are focusing on TAM reprogramming, termination of macrophage recruitment, and interference of TAM survival (73). However, the established method counting on the blockade of the CSF1/CSF1R axis would inevitably impair tissue-resident macrophages, leading to obscurity in efficacy (74). Moreover, bare drug delivery to macrophage is often with “off-target” effects and delivery barriers are still high (75).

Selenium deficiency was found to be associated with the inhibited phagocytosis of mouse macrophages due to the accumulation of oxygen free radicals and weakened antioxidant capacity (76). Chitosan oligosaccharide-conjugated selenium treatment could effectively elevate phagocytosis and increase the secretion of anti-inflammatory cytokine in mouse peritoneal macrophages, highlighting this kind of selenium compound as potential food ingredient in cancer prevention (77). Methylseleninic acid (MSeA), a selenium donor, blocked the M1 polarization of *Mycobacterium tuberculosis* (MTB)-infected macrophages through the induction of both canonical autophagy and LC3-associated phagocytosis (LAP), indicating that selenium had a restricting function against intracellular MTB by the host immunological response of macrophages (78). These results collectively suggest that selenium is a critical regulator of macrophage immunological responses.

**Figure 1:**
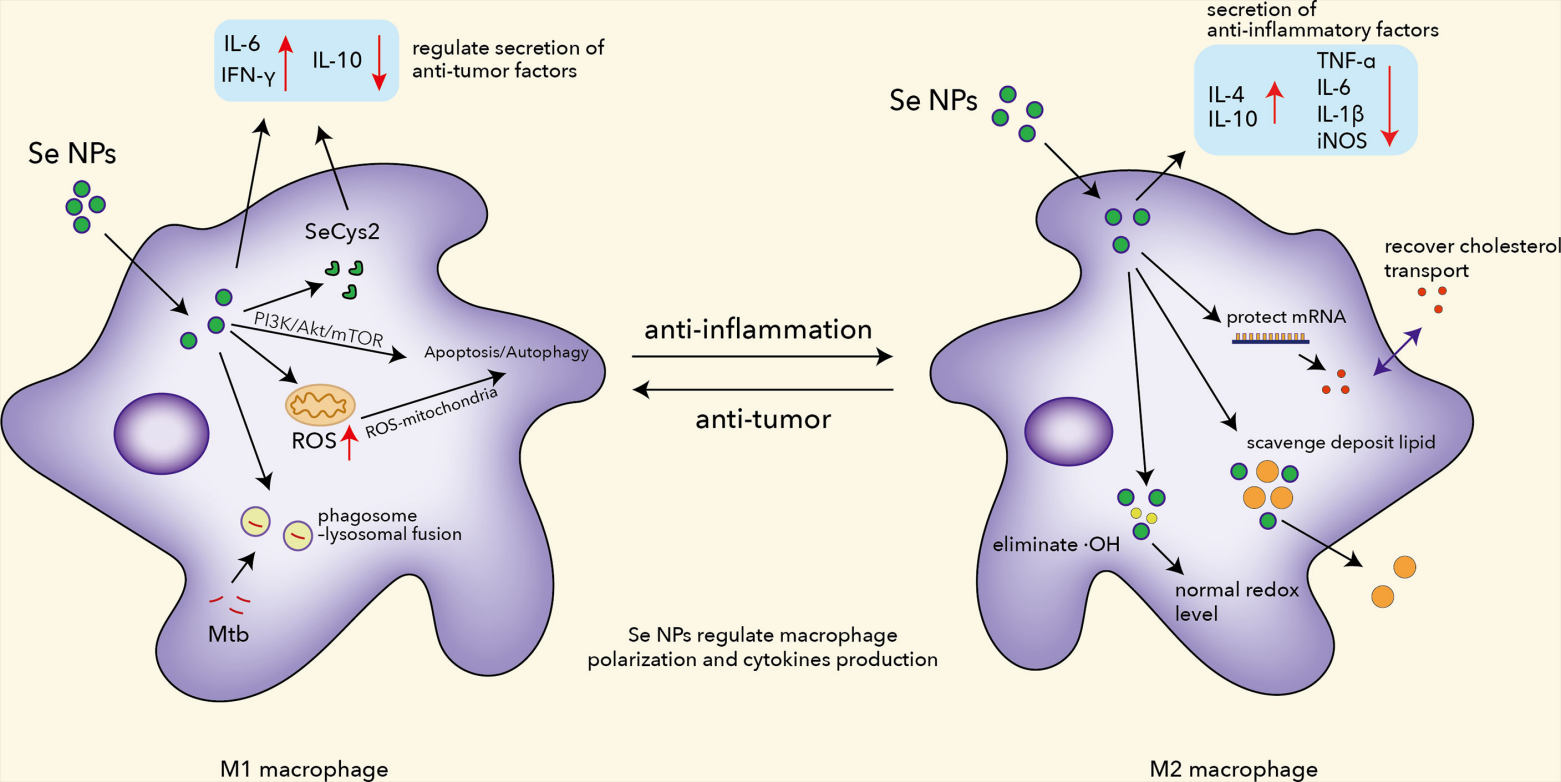
The functions of Se NPs to program macrophage immune responses, including regulating polarization, ROS production, apoptosis and autophagy induction, Mtb clearance, mRNA protection, and lipid scavenging.

Recently, nanoparticles involved in immunotherapy have shown alluring prospects in programming macrophages. Iron oxide nanoparticles have been reported to induce M1 macrophages *in vitro* and inhibit mammary tumor growth *in vivo*, with extra discovery of inhibition of metastases in the liver and lungs (79, 80). Similarly, Se NPs have found great impact on macrophage programming due to the vital role of Se in the immune system (**Figure 1**). Se NPs have been proved to successfully induce M1 macrophage polarization, which would further promote the antitumor activities of macrophages (81). Moreover, they significantly elevated the secretion of antitumor factors such as IFN-γ, to further enhance the antitumor activities and antigen presentation of macrophages (82). Se NPs could also induce a higher level of ROS, as well as adhesion, phagocytosis, fusion, and receptor profiling such as ICAM-1, CD47, and CD172α in TAMs to improve antitumor activity (83). Additionally, Se NPs could ameliorate macrophage infiltration as evidenced by decreased CD68 levels in colon tissue sections (84). The anti-inflammatory effects of Se NPs were found to involve the modulation of cytokines including IL-6 and TNF-α. Overall, Se NPs could inhibit the activation of macrophage by suppressing the nuclear translocation of NF-κB to alleviate inflammation.

Malignant pleural effusion (MPE) is considered a dangerous situation in lung cancer progression, characterized by the presence of metastasis or malignant cells. Two major problems are poor drug delivery to floating malignant cells and an immunosuppressed environment which urge novel therapeutic strategies. Song et al. introduced Se NPs with lentinan (LNT) for immunotherapy, which displayed excellent effects in treating MPE (85). Absorbed by macrophages, Se NPs@LNT partially transformed into SeCys_2_, downregulating the secretion of inflammatory and immunosuppressed cytokines. Se NPs@LNT appeared to have great potential to depolarize M2 macrophages into the M1 phenotype, blocking protective effects for cancer cells provided by the M2 macrophages. Wang et al. introduced a kind of immunogenic core–shell Au@Se NPs by a gold–selenium coordination bond to realize nanoparticle-mediated local photothermal-triggered immunotherapy (86). The combination of SeNP-mediated chemotherapy and AuNS-induced photothermal therapy not only generated a localized antitumor-immune response with excellent cancer killing effects under the presence of tumor-associated antigens but also effectively reprogrammed the TAMs from M2 to M1 phenotype with tumoricidal activity to devour distant tumors. All this evidence indicated that Se NPs are a potential therapeutic candidate for cancer immunotherapy by modulating macrophage functions.

Selenium levels are closely related to the activation of macrophages and phenotypic switch (M1 type to M2 type) and can also directly regulate the migration and phagocytosis functions of macrophages (39). The immune escape of Mycobacterium tuberculosis (Mtb) has been a long-term issue that hinders the eradication of the pathogens, which therefore contributes to the deadly TB disease. It has been proved that serum selenium is lower in TB patients as compared with health volunteers, which indicates the potential roles of selenium in the development of TB (87). Additionally, selenium showed a significant increase in levels of immunoglobulin and leukocyte count in patients after treatment as compared to the control group, which indicated the promising functions of selenium in oxidative and immune regulations in TB patients (88).

Macrophages are the most common host cells of Mtb in pulmonary TB, which can therefore serve as a target for TB treatment. Taking the advantages of nanotechnology, Se NPs also possessed an outstanding ability to modulate macrophage functions against TB. There are two critical pathogenesis hallmarks for TB: the escape of Mtb from lysosome destruction and the limited drug delivery into the Mtb-infected macrophages, which contribute to the 6–12-month-long TB therapy involving multiple drugs that always cause side effects and drug-resistant mutants (89–91). Aiming to solve these issues, our group has previously introduced a kind of macrophage-targeted Se NPs to promote the chemotherapeutic and lysosomal killing efficiency of macrophage against intracellular Mtb (92). By loading Se NPs with isoniazid, these Se NPs were further modified by mannose to achieve an Ison@Man-Se NP system for macrophage targeting by the mannose receptor (CD206) on the macrophage surface. The innovatively synthesized and characterized Ison@Man-Se NPs showed strong bacteriostatic and bactericidal effects against extracellular Mtb. Ison@Man-Se NPs could preferentially enter macrophages and accumulate in lysosomes to speed up the release of isoniazid for enhanced intracellular Mtb killings. Moreover, we also illustrated that Ison@Man-Se NPs induced antimicrobial immunity in macrophages, which include the promotion of phagosome–lysosomal fusion of Mtb, autophagosome lysosome sequestration/destruction of Mtb, autophagy/apoptosis induction through ROS-mitochondria and PI3K/Akt/mTOR signaling, and anti-TB polarization. By manipulating these macrophage immunological responses, Ison@Man-Se NPs could combine the immunological killing effects with the targeted drug killing effects to achieve a more effective intracellular Mtb clearance. These results strongly suggested the potential to enhance anti-TB treatment efficiency by manipulating the immunological responses of macrophages.

Except for facilitating the phagolysosome and polarization function of macrophages, Se NPs can also control inflammatory responses by regulating macrophages. Previous work has proved that Se NPs could downregulate the expression of pro-inflammatory factors like TNF-α, PGE2, and TBAR (93). Rheumatoid arthritis (RA) is a chronic autoimmune disorder that affects joints primarily, with deterioration of the eyes, skin, kidneys, and other organs (94). In the progression of RA, macrophages are recruited to secrete pro-inflammatory cytokines and ROS as pro-inflammatory phenotypes (95). Alleviation of joint inflammation has become the core goal of RA treatments, where macrophages serve as important target cells during inflammation. Traditional treatments usually relied on disease-modifying antirheumatoid drugs (DMARD), which currently include conventional synthesis DMARDs (methotrexate, leflunomide, etc.) and biological DMARDs (TNF-α inhibitor, cytokine inhibitor, etc.). However, their low efficacy and strong side effects remain a big threat to the life quality of patients during treatment (96). To achieve better efficacy and lower drug toxicity, Zheng coated Pd@Se NPs with hyaluronic acid (HA), endowing it with a macrophage-targeting ability (95). Their results showed that Pd@Se-HA NPs powerfully eliminated ·OH due to their antioxidant property compared to Se NPs. Zheng et al. also found that Se NPs inhibited the expression of pro-inflammatory cytokines and promoted the expression of anti-inflammatory cytokines (IL-4 and IL-10) in RAW264.7 cells with entire elimination of ROS. Interestingly, Pd@Se-HA NPs have combined advantages from both Se NPs and Pd NPs, which means it benefits from immunotherapy and photothermal therapy, offering an effective solution to eliminate inflammation mediated by macrophages.

Atherosclerosis, caused by the buildup of fats, cholesterol, and other substances in and on artery walls, remains a major cause of death around the world. Accumulation of macrophages overloaded with lipids during atherosclerosis would block normal blood flow and lead to a severe consequence. To ameliorate atherosclerosis, Yang et al. have built plaque-targeted Se NPs constructed with D-mannose and calpain inhibitory peptides (CIP) as an MSeNP@CIP system (97). MSeNP@CIP could scavenge deposited lipids in macrophages and recover cholesterol transport in macrophages by protecting mRNA ABCA1 and ABCG1. It should be highlighted that MSeNP@CIP also alleviated inflammation by reprogramming macrophages from M1 phenotype to M2 phenotype with the potential of rescuing damage caused by high-fat feeding. Xiao et al. found that chitosan-modified Se NPs could inhibit the migration of vascular smooth muscle cells (VSMC) by regulating adhesion molecules, partly ameliorating deterioration (41). These findings provide more insights into the mechanisms of Se NPs against atherosclerosis and further highlight the potential of Se NP-regulated macrophage functions as a therapeutic strategy for atherosclerosis. All this experimental evidence indicates that Se NPs could serve as a kind of potent macrophage function regulator against different diseases.

## Se NPs regulate dendritic cell functions

Dendritic cells (DCs) are considered as a specific type of immune cells that connect innate and adaptive immunity, receiving signals from tissues, and presenting processed antigens to naive T cells, contributing to guiding T-cell differentiation (98). These functions place DCs at the fulcrum of the antitumor and anti-infection T-cell responses and suggest that regulating the biological activity of these cells is a viable therapeutic approach to indirectly promote T-cell response for immunotherapy. Furthermore, DC disturbance is also manifested in inflammatory and autoimmune diseases such as RA and inflammatory bowel diseases, in a sense, which infers an entry point for immunotherapy (99).

Selenium deficiency inhibits immune cell differentiation, affects immune response, and leads to cellular and humoral immune dysfunction. By feeding the chickens with a low-Se diet, Sun et al. found that selenium deficiency could inhibit the expression of selenoproteins and change the secretion of IL-10, IL-12p40, and IFN-γ and decrease the expression of cell-surface markers including CD11c, CD40, CD86, and MHC II in DCs (100). Selenium (sodium selenite) can regulate the differentiation and immune function of mice and human dendritic cells by various pathways (101, 102). By regulating selenoproteins, selenium is involved in DCs during different stages of differentiation and activation; however, how selenium regulates DC differentiation and immune function by regulating selenoproteins is still unclear.

Driven by nanotechnology, applying nanoparticles to regulate dendritic cell functions became feasible. It has been reported that modified AuNPs and multiwalled CNTs could boost the internalization of DCs and further promote the activation of T cells (103, 104). Recently, it has been reported that Se NPs@LNT could also boost the maturation of DCs in MPE (85). Cremonini et al. tested the effects of Se NPs on bacteria and DCs, which indicated that neither the biogenic nor the synthetic Se NPs affected the viability of human DCs, nor did they elicit the production of ROS or the substantial secretion of pro-inflammatory and immunostimulatory cytokines (105). These results indicate that Se NPs might have no significant effects on DC functions and also demonstrate that biogenic Se NPs are biocompatible structures that could be administered, either alone or in combination with antibiotics, in new therapeutic strategies.

**Figure 2:**
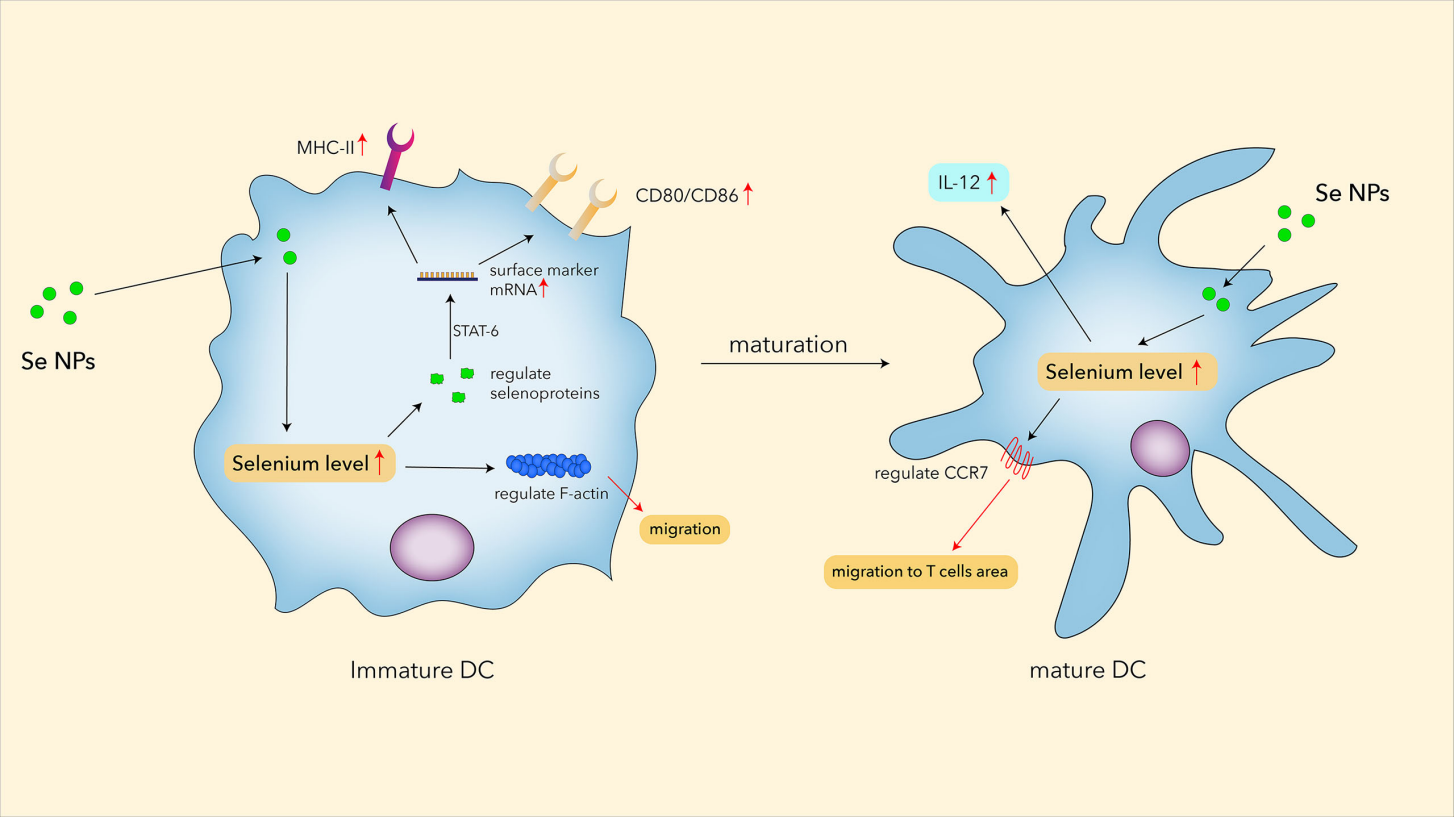
The potential roles of Se NPs in regulating functions of DC.

However, it has been proved that selenium could regulate the differentiation and immune function of human DCs by regulating the co-stimulatory molecules and cytokines through immune function activation controlled by selenoproteins (101). This study also found that 0.1 μM Se could regulate F-actin and the production of selenoproteins in immature DCs. While F-actin can impact DCs’ migration, some elevated selenoproteins, such as MsrB1, might activate the STAT6 pathway and further regulate CD80, CD86, IL12-p35, and IL12-p40 in DCs. Cytokines such as IL-12 have increased in Se NP-fed tumor-bearing mice (106). As IL-12 is mainly secreted by DCs and macrophages; the results indicated that Se NPs have the possibility to modulate the function of DCs (**Figure 2**). Wang et al. found that the combination of PTT with immunogenic core–shell Au@Se NPs could enhance the positive expression of Hsp70 in cancer cells, and Au@Se NPs could effectively stimulate the maturation of DCs with antigen-presenting functions, reminding us the potential application of Se NPs in antitumor immunity (86). Moreover, by stimulating DC maturation, PTT-combined Au@Se NPs could initiate a stronger antigen-presenting function to activate T cells for more effective antitumor treatments. However, there is still a lack of evidence to confirm that Se NPs directly enhanced dendritic cell functions. More works are needed to explore the effects of Se NPs on immunological functions of DCs.

## Se NPs enhance natural killer cell functions

Natural killer (NK) cells play a vital role in innate immune responses to cancer and infection, which is highly related to their surface receptors for recognizing tumor cells and pathogen-infected cells, leading to the activation of receptor-triggered cytotoxicity and cytokine production. In recent decades, NK cells have attracted more and more attention owing to their predominant antitumor and anti-infection properties. A previous review has concluded that the inorganic compound selenite, a derivative form of selenium, could upregulate the sensitivity of mesothelioma cells to NK cells by reducing HLA-E expression, which indicated the potential of NK cells for antitumor treatments (107). Although lots of efforts have been made, immunotherapy strategies by manipulating NK cells remain a substantial challenge.

NK cell-based immunotherapy represents a promising strategy to overcome the bottlenecks of cancer treatment. However, the therapeutic efficacy is greatly limited by downregulation of recognition ligands on the tumor cell surface. Cytotoxic effector functions in NK cells are usually limited by inhibitory signals induced by NKG2A receptor engagement (108). Adjei et al. delivered siRNA for the TGF-β receptor through a nanoparticle system to recover inhibited NK-cell functions, revealing the potential of functional nanoparticles in regulating NK-cell responses (109). Selenium-containing nano-emulsions could effectively potentiate the recognition of NK cells against cancer cells through upregulated NKG2D and NKG2D ligand expression dependent on DNA damage response pathways (110). This work not only demonstrates a simple nanoemulsion strategy to co-deliver synergistic drugs on the application and action mechanisms in NK-cell adaptive therapy against cancer but also indicates the potential of selenium-containing nanosystems for NK-cell function regulations. Moreover, the selenium-containing complex could synergize NK cells to enhance immunotherapy effects against prostate cancer by activating TRAIL/FasL signaling (111). Pan et al. also introduced pemetrexed (PEM) into a selenium-containing nanosystem, which could effectively increase the sensitivity of human NSCLC cells to NK cells by regulating pro-inflammatory cytokines such as IFN-γ and TNF-α (112). However, the exact mechanisms of selenium-mediated NK-cell immunological responses need to be further investigated.

**Figure 3:**
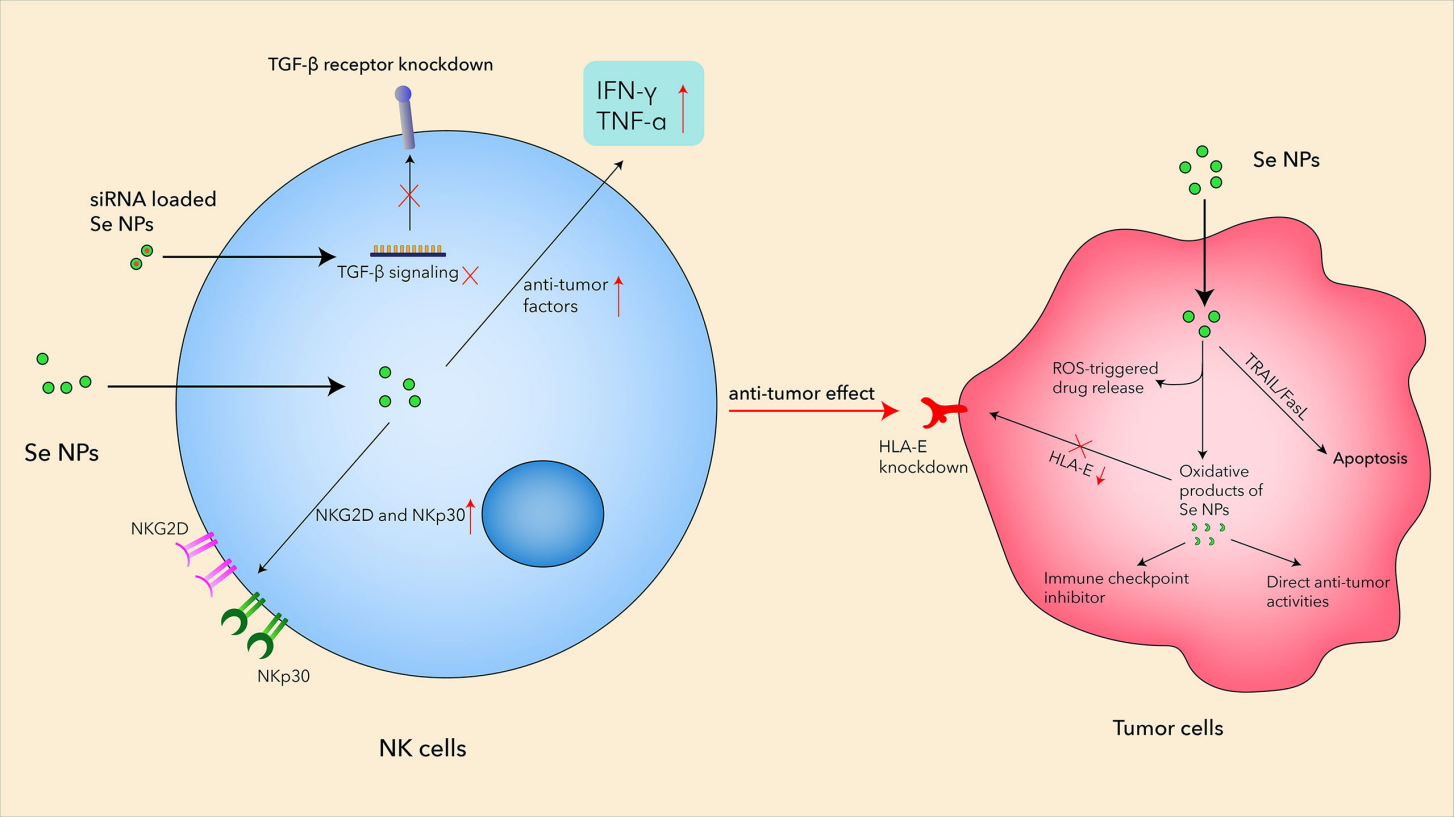
The mechanisms of Se NPs enhancing antitumor activities in NK cells.

Based on the remarkable advantages of Se NPs, more and more related therapeutic strategies have been developed to enhance NK cells. Aiming to break the blockage between HLA-E and NKG2A, Wei et al. focused on self-assembling selenopeptide nanoparticles by combining a tumor-targeting motif (RGD), an enzyme-cleavable motif (PLGVR), and a ROS-responsive motif to activate the antitumor activity of NK cells in a programmed manner (113). These self-assembled selenopeptide nanoparticles could strengthen tumor chemoimmunotherapy through the activation of NK cells mainly by the oxidative metabolite of the selenopeptide through enzyme-induced size reduction and ROS-driven desalinization (**Figure 3**). The *in vitro* and *in vivo* results proved the mutual promotion between the DOX-induced chemotherapy and the selenopeptide-induced immunotherapy by enhancing NK-cell responses, which synergistically contributed to the improved antitumor efficacy.

Natural killer group 2A (NKG2A) is an inhibitory checkpoint receptor that binds human leukocyte antigen-E (HLA-E) expressed on cancer cells, which could lead to the elimination of cancer cells by NKG2A-positive NK cells. However, the systemic administration of the anti-NKG2A antibody could also increase NK cell-mediated cytotoxicity to HLA-E-expressing normal cells, which therefore requires a more effective strategy to boost the anti-NKG2A immunotherapy. Gao et al. reported a nanomedicine, called PSeR/DOX, that comprised a tumor-targeted radiation-sensitive diselenide-containing system with chemotherapy drug encapsulation (114). The oxidation product of the above Se-containing NPs could function as an immune checkpoint inhibitor with both direct antitumor effects and immunomodulatory activity in lung metastatic and subcutaneous tumor models (**Figure 3**). Unlike traditional drug delivery systems, Se-containing NPs not only have a sensitive response to radiation stimuli but also possess the potential anticancer effects and immune checkpoint inhibitor activity with radiotherapy. These results suggested that Se NPs could be loaded with different anticancer agents and further combine with immunotherapy and radiotherapy to achieve more effective antitumor efficiency with reduced side effects.

NK cells also play important roles in the malignant pleural effusion (MPE), which remains a treatment bottleneck in advanced lung cancer, due to its complicated microenvironments and “cold” immunity. Song et al. prepared translational selenium nanoparticles coated with immune-modulating macromolecule lentinan (Se NPs@LNT), which were designed to restore the dysfunctional immune cells in a patient-derived MPE microenvironment (85). The Se NPs@LNT-based nanotherapeutic strategy could potentiate the proliferation of NK cells with increased NKG2D and NKp30 expression, indicating the activation of NK cells after Se NP treatment (**Figure 3**). This study established a new translational Se NP-based nanotherapeutic strategy for MPE linked to lung cancer, operating by converting “cold” MPE to “hot” MPE with the assistance of enhanced NK-cell responses by Se NP treatment.

## Se NPs regulate T-cell activities

T cells are one of the most essential white blood lymphocytes which play important roles in the adaptive immune responses and participate in the protection effects of the body against infection, cancer, and inflammatory and other chronic diseases. Several types of T cells have been intensively studied in recent decades, including helper, regulatory, cytotoxic, and memory T cells, which dramatically enhance our understanding of T-cell immunity. Based on the critical roles of T cells against cancer and infections, T cells have been developed as targets into different immunotherapy strategies, such as PD1/PD-L1 therapy and CAR-T therapy. Up to now, T cells are the most widely used immune cells that have been permitted for clinical immunotherapy, especially in cancer immunotherapy. However, there are still some remaining issues that need to be further explored, such as the low efficiency, off-target effects, and high costs.

Selenium has attached importance due to its particular position in human health as a trace element. In recent decades, several pieces of research have uncovered the relationship between selenium and T cells. The deficiency of selenium led to atrophy of the thymus, spleen, and lymph in mice, where the CD3+ and CD8+ populations in these mice were significantly reduced, suggesting the declined function of T cells (115). They also showed that the selenium deficiency could inhibit T-cell activation and proliferation, which indicated the critical roles of selenium in T-cell functions. Supranational selenium intake has also been shown to regulate adaptive immunity by favoring proliferation and differentiation of activated CD4-positive T cells toward Th1 cells, which plays critical roles of the T-cell responses against virus or bacterial infection (116).

The biological effects of selenium on T cells might also be exerted through the actions of selenoproteins, which exhibit a wide variety of functions within T cells that include regulating calcium flux induced by T-cell receptor (TCR) engagement, shaping the redox tone of T cells before, during, and after activation, and linking TCR-induced activation to metabolic reprogramming required for T-cell proliferation and differentiation (117). Additionally, selenoproteins have a major role in suppressing ROS production in T cells and the loss of selenoproteins would result in T cells being defective in TCR-induced and ROS-sensitive responses, indicating the essential role of selenoproteins in T-cell proliferation in response to TCR stimulation (115).

**Figure 4:**
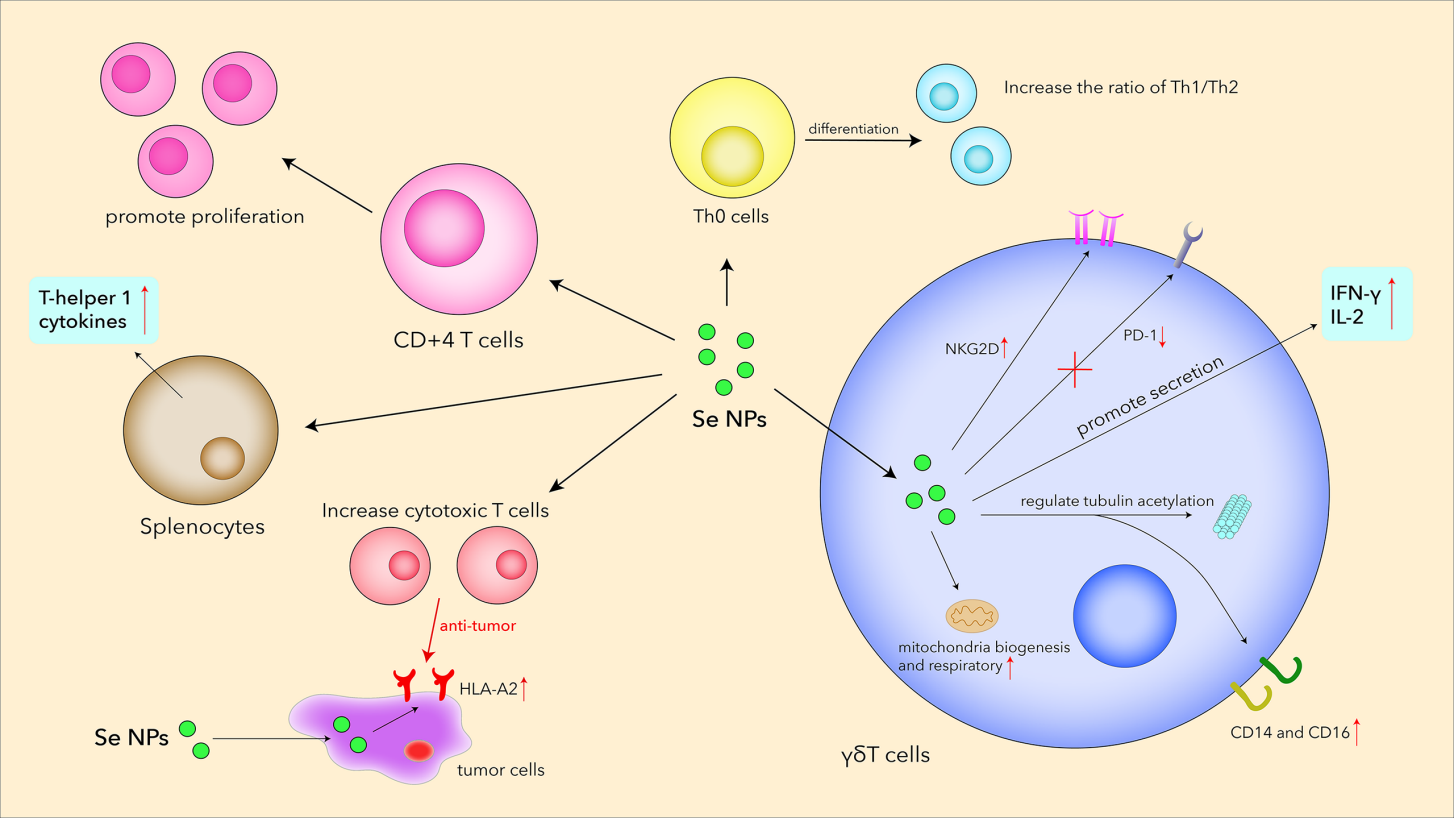
Se NPs promote T-cell proliferation, differentiation, secretion, receptor expression, and cytotoxicity.

In the last few years, Se NPs have been proved to promote T-cell proliferation and regulate T-cell functions by acting as an immunomodulatory agent (**Figure 4**). Song et al. explored the relationship between Se NPs and T cells in MPE treatments (85), which indicated that Se NPs could dose-dependently induce CD4+T-cell proliferation. The ratio of Th1/Th2 increased as well as Tc cells and γδ T cells, inducing a higher release of IFN-α and stronger antitumor activity. Shamsi et al. found that the combination of aerobic exercise training (AET) and Se NP administration could decrease tumor volume and increase Th1 cytokines in the splenocytes of tumor-bearing mice, which also indicated the anti-tumor T-cell immune responses enhanced by Se NPs (118).

Low tumor mutational burden and absence of T cells within the tumor sites are typical characteristics of “cold immune tumors” that paralyze the immune system, which therefore derives a novel therapeutic strategy of reversing “cold tumors” to “hot tumors” infiltrating a high degree of T cells to activate antitumor immunity. Recently, an immunogenic core–shell-like nanoplatform (Au@Se NPs), with AuNSs encapsulated by Se NPs, was constructed for a potent PTT-induced immunotherapeutic strategy against tumor growth (86). The combined PTT with synthesized Au@Se NPs can trigger apparent infiltration of T cells (CD8^+^ and CD4^+^ T cells) in the tumors; systemic as well as local cytokines change toward promoting inflammation. Moreover, the synergy between SeNP-mediated chemotherapy and AuNS-induced PTT could also reduce the percentage of Tregs (CD3+CD4+Foxp3+), which was helpful for the reversing of the immunosuppressive microenvironment in tumors.

γδ T cells are T cells that express a unique TCR, which is made up of one γ chain and one δ chain. This type of T cells, situated between innate immunity and adaptive immunity, plays a prominent role in the initiation and propagation of immune responses (119). γδ T cells exhibit important roles in immune surveillance and immune defense against tumors and infectious diseases, which therefore makes them a kind of attractive effector cells for cancer immunotherapy. By secreting pro-apoptotic molecules and inflammatory cytokines, γδ T cells have been demonstrated to show strong antitumor therapy effects. Given this, Hu et al. creatively utilized Se NPs to strengthen the antitumor cytotoxicity of γδ T cells for stronger cancer killing and tumor growth inhibition efficacy (120). Se NP-treated cells showed much lower mortality than those co-incubated in Na_2_SeO_3_ with similar selenium contents, reflecting the excellent biocompatibility of Se NPs. Se NPs significantly upregulated NKG2D and IFN-γ in γδ T cells, whereas the expression of PD1 was downregulated *in vivo* for alleviating immunosuppression. These changes induced by Se NPs could further contribute to the enhancement of T-cell effector functions and inhibition of tumor growth. Additionally, Se NPs could significantly potentiate antitumor cytotoxicity of Vγ9Vδ2 T cells by regulating cytotoxicity-related molecules and tubulin acetylation. This work introduced a new strategy to enhance the antitumor cytotoxicity of human Vγ9Vδ2 T cells only by Se NP treatment, not the generally used gene modification with high costs, implicating the promising clinical perspective of Se NP-based nanotechnology in γδ T-cell immunotherapy for malignant tumors.

## Other immunotherapy strategies with Se NPs

In recent years, Se NPs have exerted superb potential properties in immunotherapy by enhancing immune responses, upregulating the expression of cytotoxicity-related molecules and inducing apoptosis and autophagy (28). Intriguingly, more positive functions have been discovered in other immunotherapy strategies driven by Se NPs. Cytokine-induced killer (CIK) cells, defined as heterogeneous cells comprising CD3+CD56- T cells, CD3-CD56+ NK cells, and CD3+CD56+ natural killer T (NKT) cells, are also one of the hottest strategies that possess the potential to combat cancer by immunotherapy. It is a novel tool that exhibits MHC-unrestricted targeting ability and is accessible to culture *in vitro*. Herein, the combination of CIK with other antitumor agents, such as checkpoint inhibitors and antibodies, is developed and is expected to achieve more effective treatments in clinic (121, 122). It is reported that CIK cells exhibit cytotoxicity mainly through interactions with members of natural killer group 2 (NKG2D), which is also seen in NK cells. Notwithstanding the therapeutic potential of CIK cells, patients who receive treatments of CIK cells still confront short persistence *in vivo*, including drug resistance and relapse issues (123).

To improve CIK cell-based immunotherapy, Liu et al. developed a synergistic strategy combining Se NPs with CIK cells and further explored the therapeutic effects on human liver cancer cells (HepG2) (124). They interestingly found that the viability of HepG2 cells dramatically decreased by joint utilization of Se NPs and CIK compared to bare CIK treatments or Se NP treatment alone. Further mechanistic studies indicated that Se NPs significantly increased the secretion of pro-inflammatory cytokines such as IFN-γ and remarkably reduced the secretion of IL-10 and TGF-β by CIK cells. Additionally, co-treatment of Se NPs with CIK cells could greatly upregulate the expression of NKG2D on CIK cells and upregulate the expression of NKG2D ligands on HepG2 cells. These excellent properties contribute to the enhanced recognition and interaction between CIK cells and HepG2, maximizing the cytotoxicity killing effects to cancer cells. Intriguingly, Liu et al. also found that Se NPs could prolong CIK cell persistence *in vivo*, mainly by upregulating the expression of IL-15, which was essential to CIK cell survival. The prolonged CIK cell persistence was also associated with the increased GSH and GSSG *in vivo*. Considering that selenium, especially in the form of selenoprotein, is quite important in cellular metabolism, more research is needed to confirm the specific functions of Se NPs on metabolism. Several metabolic products, such as SeCys2, can promote CIK cells in cancer cell clearance. A challenge to kill tumor cells is that they could induce ER stress in immune cells and achieve immune escape. Se NPs could effectively downregulate the expression of ER stress marker-C/EBP homologous protein (CHOP) in CIK cells, whereas Se NPs evoked the generation of ROS in tumor cells, further inducing tumor cell apoptosis.

Diabetes mellitus (DM) is a major chronic disorder worldwide, which is commonly characterized by the abnormally high blood sugar (glucose) levels. Especially type 2 DM, characterized by dysfunction of pancreatic β-cells and low insulin sensitivity, has become the most extensive DM that would further cause some health problems as heart disease, kidney disease, and stroke. Se NPs have been proved to show antidiabetic activity manifest as increasing body weight, decreasing blood glucose and insulin levels, reducing serum lipid levels, and improving antioxidant status in mice (125). The oral administration of Se NPs resulted in a significant amelioration of the levels of serum fasting blood glucose, insulin, IGF-1, AST, ATL, and CK-MB as compared with STZ-induced diabetic rats (126). Mohamed et al. also proved that the synergistic use of CTS-Se NPs and metformin could alleviate cardio-hepatic damage in a type 2 diabetes mellitus model *via* regulation of caspase, Bax/Bcl-2, and Fas/FasL-pathway with reduced lipid accumulation and pro-inflammatory cytokine levels and restored antioxidant capacity. These findings confirmed the antidiabetic effects of Se NPs, which might be associated with the systemic immunological regulation effects of Se NPs. However, more detailed works are needed to further explore the immunological regulation mechanisms for the *in vivo* antidiabetic effects of Se NPs.

## Conclusion and future perspective

In recent decades, selenium has been placed with great expectation in treating several kinds of intricate diseases, for its promising effect on anti-oxidation, hormonal regulation, and immunoregulation effects. Selenium is a well-known component of many enzymatic proteins (selenoproteins), including GPx (6). GPx is one of the most important enzymes that neutralize the effect of ROS and phospholipid hydroperoxides to maintain oxidation–reduction (redox) balance in cells (127). Appropriate concentrations of selenium exhibit considerable antioxidant and immunological regulatory properties, which is therefore considered to be a potential therapeutic agent in various diseases. Nevertheless, the narrow safety dosage of selenium compounds still limits their biological application as the excess dosage of selenium would result in unexpected adverse effects.

**Figure 5:**
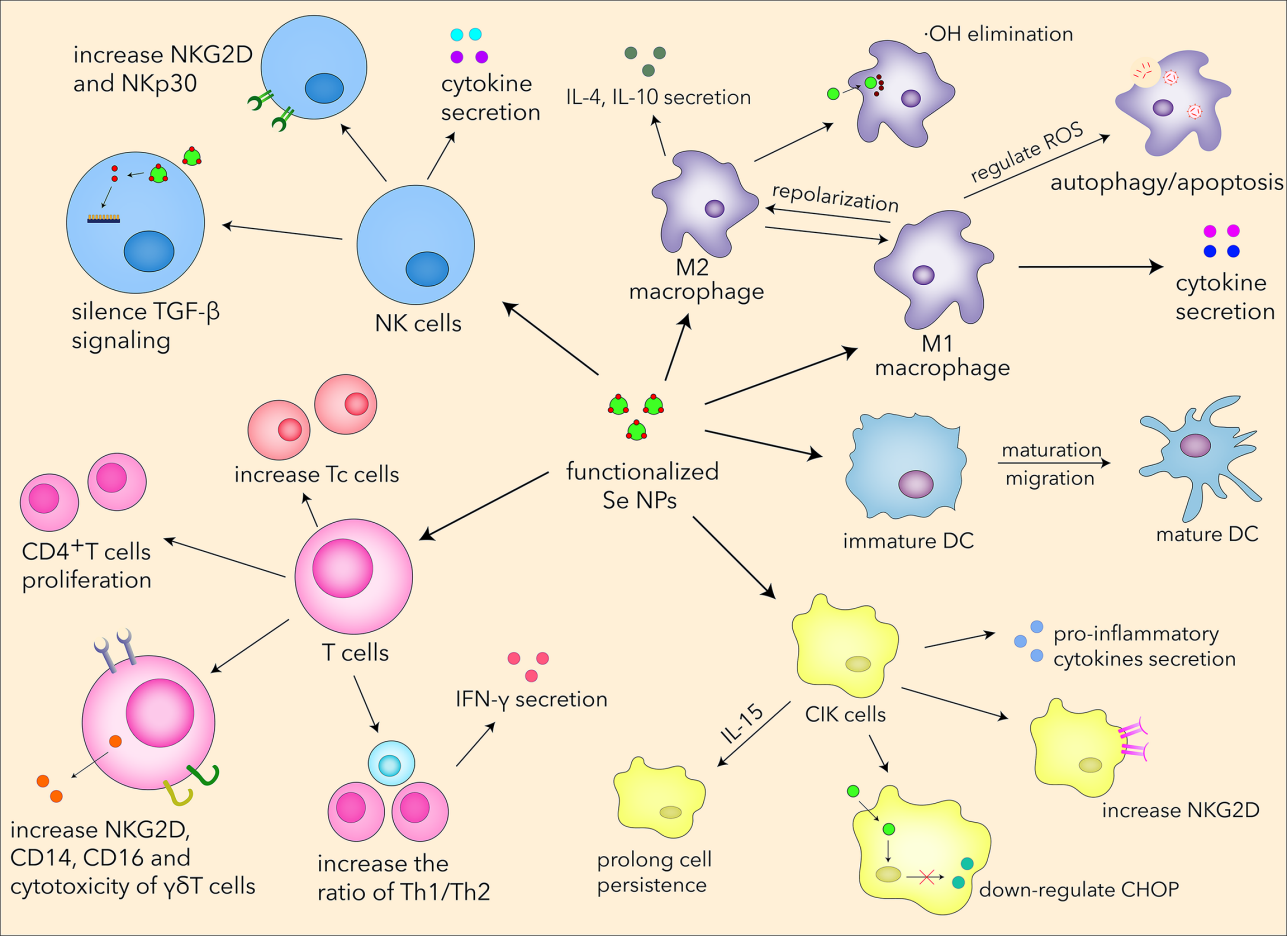
The systematic immunological regulation effects of Se NPs.

In view of the essential role of selenium in human diseases, we summarized the basic biological functions of Se NPs. Se NPs could regulate intracellular ROS levels, which can control the oxidative stress to protect normal tissues and cells during chemotherapy. Se NPs also revealed great anti-inflammatory activities through reprogramming immune cells and downregulating pro-inflammatory molecules. Currently, the carrier roles of Se NPs have shown increasing attention for drug and gene delivery. With specific surface modification, Se NPs could successfully deliver chemotherapeutics or nucleic acids to cancer cells with high tumor-targeting effects. Interestingly, although Se NPs served as antioxidative agents in normal tissues and cells, it can also dramatically upregulate the ROS level in diseased cells. Based on this outstanding property, Se NPs could induce apoptosis and autophagy, which is regarded as one of the most important properties of Se NPs for cancer cell inhibition or intracellular pathogen killings. Taking the advantages of these biological functions, Se NPs have been expected to serve as a novel nano-agent to support immunotherapy. As shown in **Figure 5**, we summarized the current understanding for the systematic immunological regulation effects and mechanisms of Se NPs.

As the first part of the body to protect us from invaders such as viruses, bacteria, parasites, and toxins, or to sense wounds, trauma, and tumors, the innate immune system utilizes some important effector cells, such as macrophages, to eliminate or rescue diseased and damaged cells. The regulatory function of macrophage is closely related to its phenotypes, which drive different results in antitumor, antimicrobic, and inflammatory responses. Se NPs possess some biological functions in immunotherapy that are poor or absent in traditional selenium compounds and have been proved to effectively impact macrophage phenotype switch, usually from M2 type to M1 type, exerting potential antitumor and antibacterial properties. In theory, intracellular Se NPs can be partially transformed into SeCys_2_, which therefore restore macrophage immunological responses by downregulating immunosuppressed cytokines. The ability of Se NPs taken into the manipulation of macrophages for disease treatment is also closely associated with intracellular ROS, which play very important roles in different diseases. Therefore, in some important diseases that are associated with ROS and inflammation in macrophages, such as tumor, rheumatoid arthritis, tuberculosis, and atherosclerosis, Se NPs are expected to enhance the immunological protection effects of macrophages. As another important part of innate immunity, DC plays a special role in immunotherapy strategy developments, for its properties of activation and regulation of T cells. Despite that Se NPs have been proved to promote the maturation of DCs by combing with other materials, direct effects are barely found in current studies to prove that the single uses of Se NPs are effective for DC function manipulation. The interactions between Se NPs and DCs are still poorly understood, which also require more attention to be paid for the future application of Se NPs.

In recent years, NK cells have attracted much attention in immunotherapy owing to their antitumor and anti-infection properties. Se NPs have been applied to regulate NK-cell functions by serving as drug and gene carriers for the targeted modulation of surface receptor and promotion of cytokine secretion, which could finally enhance the cytotoxic effects of NK cells. Considering that selenium can significantly influence the metabolism, proliferation, and differentiation of T cells, some efforts have also been put into the regulation of T cells by Se NP treatment. As a kind of novel immunomodulatory agents, Se NPs could enhance the immunological responses of different T cells, such as Th1 cells (CD4+), cytotoxic T cells (CD8+), and γδ T cells (85), therefore manipulating adaptive immunity for disease treatment. It is attractive that Se NPs can also optimize the CIK strategies and immunity-associated diabetes therapeutics with low cytotoxicity and excellent efficacy. More interestingly, Se NPs could also regulate PD1 molecule expression in T cells, which is expected to benefit the current PD1 immunotherapy. All this evidence supports the fact that Se NPs can enhance innate and adaptive immunity by regulating multiple immune cells, which precisely contributes to novel immunotherapy strategies.

Due to attractive physical, chemical, and biological properties, we also see the potential of Se NPs in the current epidemic of COVID-19, the highly contagious and deadly disease caused by the severe acute respiratory syndrome coronavirus 2 (SARS-CoV-2). Early detection for the virus is of vital importance for the timely treatment and control of the infectious epidemic. Considering that Se NPs can serve as qualitative probes, a lateral flow kit based on Se NPs for COVID-19 has been developed with the advantages of high sensitiveness and convenience (128). Since selenium has been found to combat virus such as hepatitis C virus (HCV), human immunodeficiency virus (HIV), and influenza virus, there is a possibility to take Se NPs into treatments in COVID-19 (129–131). In consideration of the ability for selenium to strengthen the host response of the influenza vaccine by eliciting antibody production, it also provides the possibility to enhance immunization of SARS-CoV-2 (132). Moreover, ebselen, an organic selenium compound, is able to block SARS-CoV-2 replication in infected Vero cells as a potent inhibitor (133). According to this selenium-related antivirus evidence, Se NPs showed huge potential to treat COVID-19. However, the immunological regulation effects of Se NPs against SARS-CoV-2 infection are still poorly understood; more experiments should be carried out to carefully confirm the role of Se NPs in COVID-19 patients (134).

Overall, Se NPs have demonstrated tempting potentials in immunotherapy; notwithstanding, several tricky challenges should be mentioned and fully discussed. One of the most urgent challenges is their biocompatibility, which remains a tough obstacle for further clinical use. It was reported that the toxicity of Se compounds depends on animal species (135). Apart from some occasional cases of overdose where people have ingested wrongly formulated supplements (136), the effects of excess selenium in health are mostly observed in randomized controlled trials, which accurately reflected that exposure of selenium toxicity outbreak could result in long-term/permanent adverse health effects. Some previous results have demonstrated that excess selenium would introduce some health risks such as the increased risks of selenosis, alopecia, dermatitis, cancer, and type 2 diabetes (137–139). Thus, it is also very important to control the intake of selenium by humans. Although Se NPs have been proved to show relatively lower toxicity and higher degradability, the degradation and metabolism processes of Se NPs *in vivo* have not been well explored. Concerns should be paid to the degradation products on how they generate, eliminate, and influence normal tissues and cells *in vivo*, on account of excessive selenium causing toxic symptoms. The long-term use of Se NPs in clinical practice also depends on their persistence and degradation, in order to prevent chronic toxicity to the human body. Thus, to discover the degradation and metabolism processes is very urgent and would be helpful for their clinical use.

Another critical challenge is that the mechanisms of Se NPs to enhance the innate and adaptive immunological responses have not been clearly uncovered. The unknown mechanisms of Se NP-regulated immune cell functions became one of the most important restrictions of Se NPs for clinical uses as modern medicine requires the exact mechanism of drugs for disease treatment. Except for the immunotherapeutic application, some researchers have pointed out that Se NPs could serve as adjuvants to modulate immunity for vaccine development (140), while the interaction mechanisms need to be further explored.

As the most important advantages of nanotechnology, the chemical or biological functionalization of nanomaterials is expected to endow these nanomaterials to show more potent biological functions. For example, with specific surface ligand modification, nanomaterials can show enhanced target effects against target cells. Moreover, with functionalization or conjugation with other nanomaterials, Se NPs can also combine the native ability of other materials to show synergistic therapeutic effects for more effective immunotherapy strategy development. These functionalization methods would finally benefit more effective immunotherapy strategy development on the basis of Se NPs.

Currently, most of the studies about Se NPs are still restricted in laboratory studies, which have shown the strong potentials of Se NPs for biological application. Moreover, based on these laboratory results, some healthcare products about Se NPs have been industrially produced and approved for selenium supplementation and healthcare treatments. However, the industrial preparation line of Se NPs for such biological uses is still not well established; more attention should be paid to establish the intact industrial production line and create the international quality standards for these Se NP products, which is expected to further support the future potential uses of Se NPs in immunotherapy.

Although some important reviews have been made for the different roles of Se NPs, including their potentials in cancer gene and drug delivery (141), the roles of nano-selenium in health and environment (142), their biomedical applications (143), and their chemopreventive mechanism of actions (144), we believe this is the first review that systemically summarizes the immunomodulatory regulation roles of Se NPs. By discussing the immunological regulation effects of Se NPs in different immune cells and different disease conditions, we concluded that Se NPs have strong potential to be developed into a more effective disease treatment as a novel therapy strategy. Additionally, we also introduce the perspective of Se NPs for the potential immunotherapy in the future, as well as their advantages and restrictions.

Considering the limited clinical benefits of individual approaches against malignancy, it is also worthy to note that Se NP-based immunotherapy may be difficult to achieve high treatment efficiency alone; however, it has shown attractive potentials to show much higher efficiency by combining with other therapeutic strategies, such as chemotherapy and radiotherapy. Taking the advantages of drug encapsulation and delivery, Se NPs can increase the tumor target effects of chemotherapeutics for more effective tumor inhibition, which can be further enhanced by the immunological antitumor effects of Se NPs. Moreover, the killing efficiency of Se NP-delivered antibiotic against intracellular pathogens in macrophages can also be enhanced by the immunological regulation effects of Se NPs in macrophages. Moreover, Se NPs can also act as a radiosensitizer for cancer treatment, and the immunological regulation effects of Se NPs can be combined with radiotherapy for a more effective therapy. Therefore, Se NPs are expected to be developed into novel adjuvant therapy strategies that enhance the efficiency of current therapeutics. More works should be done to explore the combination of Se NP-based immunotherapy strategies with other therapeutics, such as chemotherapy or radiotherapy, that might provide new possibilities to achieve much better treatment efficiency and finally benefit the clinical therapeutics.

## Author contributions

GC and FY drafted the manuscript, contributed equally to this work, and share the first authorship; SF, HJ, KL, XL, G-BL, and JL helped to revise the manuscript; JZ, J-FX, and JP were responsible for leading this work and revising the manuscript. All authors contributed to the article and approved the submitted version.

## Funding

This work was supported by the Open Research Fund of Songshan Lake Materials Laboratory (2021SLABFN10), the Natural Science Foundation of Guangdong Province (2022A1515011223 and 2022A1515010525), the Project of Educational Commission of Guangdong Province of China (2021KTSCX038), the Guangdong Basic and Applied Basic Research Foundation (2020A1515010283 and 2021B1515140068), the National Natural Science Foundation of China (81870016), the Medical Scientific Research Foundation of Guangdong Province (A2018434), the Science and Technology Project of Dongguan (20211800904782, 202110571025), the Key Project of Science Foundation of Guangdong Medical University (GDMUZ2019005), and the Discipline Construction Project of Guangdong Medical University (4SG21279P), Hospital Fund for The First Dongguan Affiliated Hospital of Guangdong Medical University (4SG21229GDGFY01 and PU2022002).

## Acknowledgments

We tender our apologies to those authors whose deserving research was not cited in this manuscript.

## Conflict of interest

The authors declare that the research was conducted in the absence of any commercial or financial relationships that could be construed as a potential conflict of interest.

## Publisher’s note

All claims expressed in this article are solely those of the authors and do not necessarily represent those of their affiliated organizations, or those of the publisher, the editors and the reviewers. Any product that may be evaluated in this article, or claim that may be made by its manufacturer, is not guaranteed or endorsed by the publisher.
